# Selecting Appropriate Sarcopenia Screening Methods for Asian Populations

**DOI:** 10.3390/jcm9082333

**Published:** 2020-07-22

**Authors:** Yu-Ching Lin, Yi-Chien Lu, Fang-Ping Chen, Ying Chin Lin, Yun-Chung Cheung, Wing P. Chan

**Affiliations:** 1 Department of Medical Imaging and Intervention, Chang Gung Memorial Hospital, Keelung and Chang Gung University, Taoyuan 33001, Taiwan; yuching1221@gmail.com; 2 Keelung Osteoporosis Prevention and Treatment Center, Keelung 200131, Taiwan; fangping@cgmh.org.tw; 3 Department of Radiology, Wan Fang Hospital, Taipei Medical University, Taipei 116, Taiwan; 106263@w.tmu.edu.tw; 4 Department of Obstetrics and Gynecology, Chang Gung Memorial Hospital, Keelung and Chang Gung University, Taoyuan 33001, Taiwan; 5 Department of Family Medicine, Wan Fang Hospital, Taipei Medical University, Taipei 116, Taiwan; greening1990@gmail.com; 6 Department of Family Medicine, School of Medicine, College of Medicine, Taipei Medical University, Taipei 10001, Taiwan; 7 Department of Medical Imaging and Intervention, Chang Gung Memorial Hospital at Linkou, Institute for Radiological Research, Chang Gung University, Taoyuan 33001, Taiwan; alex2143@ms33.hinet.net; 8 Department of Radiology, School of Medicine, College of Medicine, Taipei Medical University, Taipei 10001, Taiwan; 9 Medical Innovation Development Center, Wan Fang Hospital, Taipei Medical University, Taipei 116, Taiwan

**Keywords:** Asian, body composition, dual-energy X-ray absorptiometry (DXA), handgrip strength, sarcopenia, adjusted skeletal muscle index

## Abstract

We aimed to determine the most appropriate sarcopenia screening method for Asian populations. We retrospectively studied the physiological differences between the sexes in healthy individuals and prospectively compared using skeletal muscle mass versus handgrip strength (HS) to screen for sarcopenia in a community-based population. Skeletal muscle mass was determined using dual-energy X-ray absorptiometry. Of 5881 healthy individuals recruited, 101 were from urban populations and 349 from a community-based population. The sexes were comparable in total lean muscle mass declines after peaking around 20 years of age. An age-dependent decline in total fat mass was found only among men;a persistent increase in total fat mass was observed only among women. The prevalence of low skeletal muscle mass significantly increased with age in both sexes only when applying the weight-adjusted skeletal muscle index (wSMI); it was significant only among men when applying the height-adjusted skeletal muscle index (hSMI). Using HS resulted in a much higher prevalence of sarcopenia in both sexes. A significant age-dependent increase in fat mass in women showed that the most appropriate adjustment method is wSMI for women and hSMI for men. Nevertheless, a primary HS survey is recommended for both sexes in Asian populations.

## 1. Introduction

Sarcopenia is an aging process related to losses in muscle mass and function; it is accompanied by reduced physical capabilities, deteriorated quality of life, and even early death. Not only does it increase national medical costs, it is considered one of the most important public health issues in geriatric care [1,2,3]. Thus, early detection of sarcopenia followed by adequate nutritional intervention is essential for improving geriatric care [1,2,3]. Several examinations for assessing sarcopenia are available including computed tomography, magnetic resonance imaging, bioelectrical impedance analysis, and dual-energy X-ray absorptiometry (DXA) [4]. Of these, DXA is widely accepted for diagnosing the prevalence of sarcopenia because of its ease of use, noninvasiveness, low cost and radiation dose (0.96–1.92 mSv) [5,6], and ability to measure lean mass, fat mass, and bone mineral density across the entire body or in specific body regions.

Sarcopenia is commonly defined as the presence of a relatively low appendicular skeletal muscle index (ASMI) and a relatively low handgrip strength (HS) when compared to those of young referents [7,8]. However, some concerns about these screening methods for sarcopenia remain. For one, normalization of the cut-off value for the ASMI in a young referent is commonly performed using either a weight-adjusted skeletal muscle index (wSMI), described by Janssen et al. [9,10], or a height-adjusted skeletal muscle index (hSMI), described by Baumgartner et al. [11]. These two adjustment methods can provide different results for the prevalence of sarcopenia, even when applied to the same population. Meng et al. reported that the overall prevalence of sarcopenia in a Taiwanese metropolitan population was lower when hSMI versus wSMI (5.7% vs. 9.7%) was used, and this discrepancy was overwhelming among women (2.6% vs. 12.5%) [12]. This has led to some concern about selecting the best method for adjusting ASMI in Asian populations. Although the Asian Working Group Society recommends using hSMI over wSMI, the prevalence of sarcopenia using hSMI with Asian women is extremely low and does not appear to increase with age [13]. The 2019 revised European Working Group on Sarcopenia has changed the primary screening tool to HS from ASMI [14]; whether this rule fits well in Asian populations remains unclear.

A competent appendicular skeletal muscle index adjustment method should equip the clinician with the ability to detect low skeletal muscle mass in an individual and also provide good agreement between the incidence of sarcopenia and the degree of difference in body composition. However, physiological differences can be found between the sexes, and these differences can significantly influence the prevalence of sarcopenia depending on adjustment method. Therefore, it is vital to understand the differences in total body composition (TBC) in healthy controls and then select the most appropriate adjustment method for each sex.

The aim of this study was to understand the physiological differences in TBC between the sexes in healthy individuals, elucidating the most appropriate method of adjusting ASMI for each.

## 2. Materials and Methods

### 2.1. Study Design

This study was divided into two parts. First, a healthy control population was used to investigate the physiological differences in TBC between the sexes and then determine the cut-off value for each adjustment method. Second, the sarcopenia screening abilities of ASMI and HS were compared in urban and community-based populations.

### 2.2. Studied Populations

#### 2.2.1. Healthy Control Population

This part was approved by the Taipei Medical University-Joint Institutional Review Board (JIRB Number N201712053), and informed consent was waived because of its retrospective nature. We retrospectively analyzed the medical records of 6177 participants aged 20 to 90 years and who received annual health examinations and full-body DXA scans at a single medical center from 1 January 2007 to 31 December 2016. Exclusion criteria were prior diagnosis or treatment for cardiovascular disease (*n* = 53), renal disease (*n* = 10), diabetes mellitus (*n* = 58), hypertension (*n* = 216), or any malignancy (*n* = 34). Sixty-seven recruits presented more than one of these, resulting in the exclusion of 296 in total. The age distribution of this healthy control population was shown to be normally distributed based on skewness and kurtosis.

#### 2.2.2. Urban Population

This part was performed retrospectively as a review of urban participants at a single institution. This segment was approved by the same institution (JIRB Number N201712053), and informed consent was waived because of its retrospective nature. The elderly population of interest included all those aged 60 years or more who were registered as residents of the Taipei metropolitan area and received DXA scans at a single medical center from 1 January 2019 to 30 June 2019. A total 111 records were reviewed. Ten were excluded for lack of an HS measure, leaving 101 recruited to the study.

#### 2.2.3. Community-Based Population

This prospective recruitment was approved by the Chang Gung Memorial Hospital Institutional Review Board (IRB Numbers 201800598B0, 201800841B0), and written informed consent was obtained from all participants. The elderly population of interest included all those aged 60 years or more who were registered as residents of an administrative neighborhood (“Li”) of the North District, Keelung City, Taiwan. The population of this community was 82,338, and a total of 369 citizens were randomly selected and invited to participate in this study. The required examinations were incomplete for 20 invitees, and these were excluded from the study.

### 2.3. Measurements

#### 2.3.1. Anthropometric Measurements

Weight, height, and body mass index (BMI) measures were obtained. The last of these was defined as the weight in kilograms divided by the squared height in meters (kg/m^2^).

#### 2.3.2. DXA Examinations

In the healthy controls and urban populations, total body composition was determined using a Lunar Prodigy (GE Healthcare, Madison, WI, USA); however, in the community-based population, a Lunar iDXA (GE Medical Systems, Madison, WI, USA) was used. The scan mode (standard, thin, or thick) was selected automatically by the scanner software based on body size and BMI. Scans from both models were analyzed using enCORE Software, version 15 (GE Healthcare, Madison, WI, USA). All measurements were performed by experienced technicians certified by the International Society for Clinical Densitometry, and all protocols and procedures were strictly followed [15].

All participants wore light indoor clothing and removed shoes and all items that could interfere with DXA results. Each participant was positioned according to the guideline set by the International Society for Clinical Densitometry [15].

The following data were obtained from the DXA analysis: total lean mass (TLM; kg), total fat mass (TFM; kg), and total bone mass (TBM; kg). Appendicular skeletal muscle (ASM; kg), was calculated by summing the lean mass amounts of the four extremities.

#### 2.3.3. Handgrip Strength Assessment

Muscle strength was assessed using dynamometers to measure the handgrip strength of those in the urban population (Exacta™; North Coast Medical, United States) and the community-based population (EH101; Camry, China); measures were not available for the healthy controls. The maximum of 3 assessments in each hand was recorded.

### 2.4. Screening for Sarcopenia

The cut-off value for diagnosing low skeletal mass in the urban and community-based populations was acquired from young referents: healthy controls aged 20 to 29 years. Two adjustment methods, hSMI and wSMI, were used. The former was calculated as ASM/height^2^ (kg/m^2^), and the latter was calculated as (ASM/weight) × 100% [10,12]. Low skeletal muscle mass Class I was diagnosed for those whose adjusted SMIs were −1 to −2 standard deviations (SDs) of those of the young referents, and Class II was diagnosed for those whose adjusted SMIs were more than −2 SDs of those of the young referents [7,8]. Low muscle strength was defined as having an HS less than 22.4 kg among the men and 14.3 kg among the women, as proposed by Liu et al. using a Taiwanese population [16].

### 2.5. Statistics

The chi-square test was used to find significant differences between the age decades in both sexes. The two adjustment methods were compared based on the resultant prevalence of low skeletal mass for each, using a generalized estimating equations test. The previously acquired ASMI for each sex was used to compare with HS in determining the prevalence of sarcopenia in the urban and community-based populations. The urban and community-based populations were compared using a *t* test. A database was established using Excel and SPSS softwares. Statistical analyses were performed using PASW Statistics version 18.0 (SPSS Inc., Chicago, IL, USA), and when *p* < 0.05, a statistically significant difference was recognized.

## 3. Results

### 3.1. Healthy Controls

#### 3.1.1. Physiological TBC Changes

**Figure 1:**
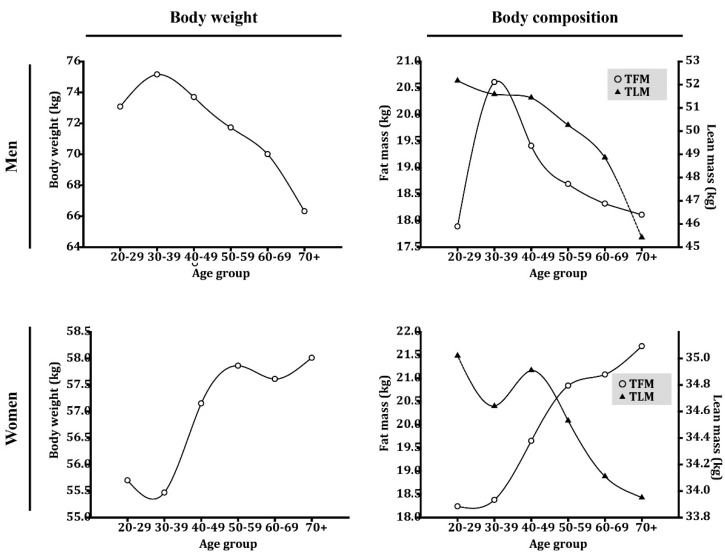
Age-based differences in body weight and body composition in a healthy control population. TFM: total fat mass; TLM: total lean mass.

Of the 5881 healthy participants, 3028 were men (mean [SD] age, 50.16 [11.27] years), and 2853 were women (mean [SD] age, 48.20 [11.52] years; Table 1). Among the men, mean (SD) body weight peaked at 75.16 (12.78 kg) at 30 years of age and showed a constant decline thereafter (Figure 1, Supplementary Table S1, Figure S1). Body composition behaved comparably, and mean (SD) total lean mass (TLM; kg) and total fat mass (TFM; kg) peaked at 20 and 30 years of age, respectively, at 52.17 (5.03) kg and 20.61 (7.71) kg, respectively. Both constantly declined after peaking. On the contrary, women’s mean (SD) body weights peaked (58.01 [8.81] kg) at a much greater age (≥70 years). Although the physiological change in mean (SD) TLM was similar in women (35.02 [4.58] kg at 20 years of age), a significant difference, compared to men, was noted in TFM. Instead of showing a constant decline in TFM starting at an early age, women showed a continuous increase in TFM from 20 to 70+ years of age, where the peak, 21.69 (6.19) kg, occurred.

#### 3.1.2. Acquiring the Cut-off Value

The young referents (*n* = 211) comprised 77 men (mean [SD] age, 26.52 [2.42] years) and 134 women (mean [SD] age, 26.66 [2.33] years; Table 1). The cut-off values for hSMI for those with Class I and Class II low skeletal muscle mass were 7.22 kg/m^2^ and 6.40 kg/m^2^, respectively, for men and 5.12 kg/m^2^ and 4.32 kg/m^2^, respectively, for women. In contrast, the cut-off values for wSMI were 30.08% and 26.51%, respectively, for men and 24.81% and 21.89%, respectively, for women.

#### 3.1.3. Prevalence of Low Skeletal Mass According to Age Decade

**Figure 2:**
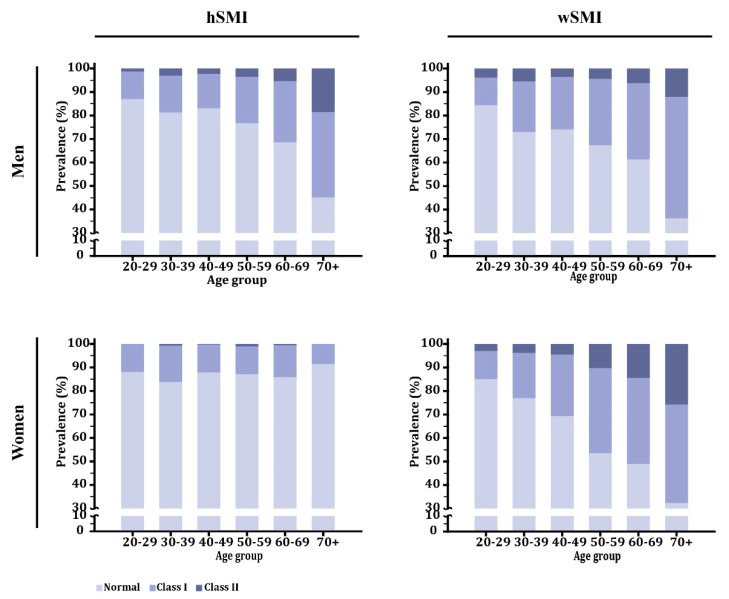
Differences in low skeletal mass prevalence by age in a healthy control population. hSMI: height-adjusted skeletal muscle index; wSMI: weight-adjusted skeletal muscle index.

Because TLM was shown to decline after 20 years of age for both sexes, the prevalence of low skeletal mass was expected to increase with age. However, only when wSMI was used to determine the prevalence of Class I and Class II low skeletal muscle mass did that bear out for both sexes (*p* < 0.001). Use of hSMI showed that the prevalence of Class I and Class II low skeletal muscle mass significantly increased with age only for the men (*p* < 0.001) but not for the women (*p* = 0.232; Figure 2, Supplementary Table S2, Supplementary Figure S1). Generalized estimating equation tests further indicated that the two adjustment methods significantly differed for the women only (*p* < 0.001) and not for the men (*p* = 0.494).

### 3.2. Urban and Community-Based Elderly Populations

In the urban population, the 101 participants comprised 51 men (mean [SD] age, 71.04 [9.68] years) and 50 women (mean [SD] age, 76.90 [11.00] years; Table 2). In the community-based population, the 349 participants comprised 117 men (mean [SD] age, 74.56 [7.10] years) and 232 women (mean [SD] age, 72.76 [7.89] years).

**Figure 3:**
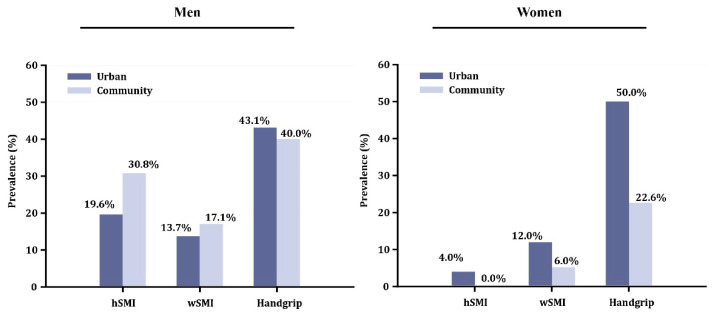
Prevalence of low muscle mass using each of two adjustment indices and of handgrip strength. hSMI: height-adjusted skeletal muscle index; wSMI: weight-adjusted skeletal muscle index.

The overall prevalence of Class II low skeletal muscle mass in the urban and community-based elderly populations using hSMI as the adjustment method was 19.6% and 30.8%, respectively, among the men and 4.0% and 0.0%, respectively, among the women. Using wSMI, those values were 13.7% and 17.1%, respectively, among the men and 12.0% and 6.0%, respectively, among the women (Figure 3, Supplementary Figure S2).

When comparing the urban elderly population with the community-based elderly population (Table 2), significantly lower TLMs were found in the latter among both the men (8.34% difference, *p* < 0.001) and the women (5.56%, *p* = 0.014). A significantly lower ASM was found only among the men (8.34%, *p* = 0.003) and not the women (4.15%, *p* = 0.148). Notably, neither BMI nor TFM differed significantly between these two populations for both sexes.

### 3.3. Handgrip Strength

When comparing the urban with the community-based elderly populations (Table 2), a significant difference in HS could not be found among the men (3.5% difference, *p* = 0.617); however, among the women, HS was significantly lower in the urban population (18% difference, *p* = 0.005). This reflected on the prevalence of probable sarcopenia in each sex when an HS cut-off is applied. The prevalence was similar between the urban and community-based men (43.1% vs. 40.0%); however, a great discrepancy was found between the women, where it was much higher in the urban population (50.0% vs. 22.6%; Figure 3).

## 4. Discussion

Our study aim was to first investigate the physiological differences in TBC in healthy controls to determine the most appropriate adjustment method for each sex, then to compare the sarcopenia screening abilities of ASMI and HS by applying them to both urban and community-based Asian populations. An age-dependent loss in TLM after peaking around 20 years of age was established across both sexes, but a significant discrepancy was found in TFM. The women, instead of showing an age-dependent loss in TFM (as shown by men), demonstrated a pronounced age-dependent increase corresponding to an increase in body weight up to the oldest age group. This rise in TFM among the older women has a substantial negative impact on studies of the prevalence of sarcopenia when hSMI is used as the adjustment method. Thus, wSMI might be the preferred adjustment method in studies of sarcopenia prevalence among women, whereas the hSMI adjustment method is more suitable for men. When both sexes are involved, HS is a more sensitive method compared to ASMI together with HS for primary screenings for sarcopenia in either urban or community-based Asian populations.

We investigated the physiological age-related differences in TBC for both sexes. Both were consistent in peaking at 20 years of age, averaging a 1% to 2% decrease in TLM every ten years thereafter until the age of 60 years was reached. Thereafter, the magnitude of TLM loss became more pronounced in the men compared to the women, and the loss can reach 7% in men (Supplementary Table S1). This pattern of change differs from what is seen in Western countries where an age-dependent loss of skeletal muscle mass starts much later (at approximately 50 years of age), and the decline in skeletal muscle mass is much greater (6.6%–23.3%) [17,18,19]. While some similarities between the sexes were found for differences in TLM, a significant discrepancy was noted in TFM. Women, instead of showing the age-dependent loss in TFM demonstrated by the men, showed an increase. Moreover, losses in TLM were overcompensated by increases in TFM, reflecting on the constant increase in body weight over time (Figure 1). The age-dependent increase in TFM among the women was primarily related to an increase in visceral fat between the 3rd and 7th decades that exceeded 400% [20]. The physiological aging process resulted in women gaining more fat compared to men and becoming more prone to obesity as well [21]. Of note, debates about the impacts of ageing and menopause on fat mass continues. A recent meta-analysis reported by Ambikairajah et al. indicates that the change in fat mass is predominantly attributable to increasing age rather than menopause [22]. On the other hand, Greendale et al. reported that gains in fat mass and losses in lean mass are the result of menopause transition-related phenomena [23]. Similarly, in our study, a drastic increase in fat mass was found among women between the ages of 40 and 59 years (Figure 1), near the menopause transition period. After this period, the rise in fat mass slowed and reached a plateau. However, another drastic increase in fat mass was found after 70 years of age, and this increase could be age-related. Thus, the net increase in fat mass could be related to both the menopause transition and ageing.

Our results indicate that wSMI might be the preferred adjustment method in sarcopenia prevalence studies involving only women, but that the hSMI adjustment method is more suitable for men. The age-dependent increase in TFM among women has a great impact on the selection of an adjustment method, as demonstrated by this study. The proposed theory was that wSMI can be affected by either a loss in skeletal muscle or a gain in fat because in both scenarios, the percentage of skeletal muscle relative to the whole-body mass is reduced [12]. Therefore, wSMI can identify overweight or obesity as sarcopenia [12,24,25]. As a consequence of age-dependent increases in TFM in healthy women, older women are more inclined to demonstrate obesity or overweight. This provides a reasonable explanation for why the wSMI adjustment method offers a significant increase in the prevalence of Class I or Class II low skeletal muscle mass as women age but that hSMI does not do so (Figure 2). To strengthen this point, we further validated these two adjustment methods in different populations. When an urban population is compared to a community-based population in the overall prevalence of sarcopenia, we anticipate that the latter would show a greater prevalence of Class II low skeletal muscle mass because comparatively, that population has a significantly lower TLM. However, use of the hSMI adjustment method cannot detect women with Class II low skeletal muscle mass in our chosen community-based population. On the other hand, use of the wSMI adjustment method showed Class II low skeletal muscle mass in 6.0% of the women in our sample, comparable to results found in other Asian studies. Of the two methods, wSMI resulted in a higher prevalence of Class II low skeletal muscle mass, the difference potentially reaching 10% [12,24,26].

In this study, two populations with differing lifestyles were enrolled. The urban population, from Taipei City, was of higher socioeconomic status than the community-based population, from Keelung City. Most urban dwellers can afford good nutrition because they have high-paying jobs; in contrast, community-based populations—those who live in more rural parts of our country—might not be able to afford good nutrition because they tend to have low-paying jobs. We observed a significant difference in mean (SD) TLM between urban and community-based populations among both men (45.54 [6.94] vs. 41.74 [5.39], *p* < 0.001) and women (34.33 [5.03] vs. 32.42 [3.90], *p* = 0.014). A recent systematic review demonstrated that as protein intake increases, the percentage of lean mass increases in older adults [27]. This can imply that the discrepancy in TLM between the two populations is a result of lifestyle differences. The discrepancy in sarcopenia prevalence found between men and women in our two populations could be related to differences in age. The men were older in the community-based population compared to the urban population, resulting in a greater prevalence of sarcopenia no matter the adjustment method applied. On the other hand, the women were older in the urban population, and the prevalence of sarcopenia was greater in the urban population no matter the adjustment method applied.

Although wSMI appears to be a better adjustment method for women and hSMI for men, HS estimates a much higher prevalence in both urban and community-based populations, and this paradox is more apparent in women. It was previously reported that HS decreased more with age than did muscle mass in women; it is reasonable to expect that elderly women can have low muscle strength without evidence of low muscle mass [28]. Auyeung et al. reported that the reduction in HS is 0.798 and 1.239 kg/year for men and women, respectively [29]. The women in our community-based population were younger overall than those in our urban population, resulting in a significantly lower HS in the latter even though the difference in ASM was not significant between the two (Table 2). The men, however, were comparatively younger in our urban population, but the two populations did not significantly differ in HS. This might suggest that HS decreases less drastically with age in men. Nevertheless, in both urban and community-based populations, HS has been proven to be a better screening tool for both sexes for the prevalence of probable sarcopenia compared to the ASMI (Figure 3). This recommendation is currently supported by the 2019 revised European Working Group on Sarcopenia, which focuses on low muscle strength as a key characteristic of sarcopenia and the use of low muscle quantity only as a confirmation of a sarcopenia diagnosis [14].

This study has a few limitations. First, this is a cross-sectional study, and no outcome data are available. Further research using a longitudinal design should be performed to clarify the outcome prediction in elderly populations. Second, the DXA machines used in this multicenter study differed; however, the same manufacturer and the same version of auto-analysis software were used when analyzing TBC, thus minimizing variations. Third, an age discrepancy was found between the urban and community-based populations in this study. However, the primary focus of this study was to validate two adjustment methods in different populations and not compare the prevalence of sarcopenia between urban and community-based populations.

## 5. Conclusions

In conclusion, the most appropriate adjustment method is wSMI for women and hSMI for men. Nevertheless, a primary HS survey is recommended for both sexes in Asian populations.

## Figures and Tables

**Table 1:** Demographic data for the healthy population.

	All Healthy Individuals		Young Referents	
	Men	Women	Men	Women
*n*	3028	2853	77	134
Age (y)	50.16 (11.27)	48.20 (11.52)	26.52 (2.42)	26.66 (2.33)
Height (m)	1.70 (0.06)	1.58 (0.06)	1.74 (0.04)	1.61 (0.05)
Weight (kg)	72.37 (10.78)	57.06 (9.03)	73.08 (11.20)	55.70 (10.78)
BMI (kg/m^2^)	25.00 (3.22)	22.85 (3.59)	24.19 (3.71)	21.60 (4.08)
TBM (kg)	2.84 (0.42)	2.19 (0.34)	3.07 (0.43)	2.30 (0.29)
TFM (kg)	19.10 (6.50)	19.96 (6.30)	17.89 (7.74)	18.24 (7.20)
TLM (kg)	50.43 (5.69)	34.61 (3.91)	52.17 (5.03)	35.02 (4.58)
ASM (kg)	22.63 (3.04)	14.56 (2.00)	24.32 (2.68)	15.28 (2.33)
hSMI (kg/m^2^)	7.81 (0.84)	5.82 (0.69)	8.04 (0.82)	5.92 (0.80)
wSMI (%)	31.47 (3.10)	25.76 (2.95)	33.65 (3.57)	27.73 (2.92)

Except for *n*, values are given as mean (standard deviation). *n*: sample size; TBM: total bone mass; TFM: total fat mass; TLM: total lean mass; ASM: appendicular skeletal muscle (sum of the lean masses of the four extremities); hSMI: height-adjusted skeletal muscle index; wSMI: weight-adjusted skeletal muscle index.

**Table 2:** Demographics of the studied urban and community-based populations.

	Men			Women		
	Urban	Community	*p*-Value	Urban	Community	*p*-Value
*n*	51	117		50	232	
Age (y)	71.04 (9.68)	74.56 (7.10)	0.022	76.90 (11.00)	72.76 (7.89)	0.014
Weight (kg)	68.01 (11.79)	62.37 (10.57)	0.002	55.88 (8.81)	54.76 (9.11)	0.428
Height (m)	1.65 (0.07)	1.62 (0.06)	0.003	1.52 (0.06)	1.52 (0.06)	0.740
BMI (kg/m^2^)	24.87 (3.90)	23.87 (3.82)	0.122	24.18 (3.45)	23.76 (3.60)	0.450
TBM (kg)	2.42 (0.41)	2.32 (0.37)	0.112	1.59 (0.24)	1.67 (0.27)	0.056
TFM (kg)	19.92 (6.03)	17.88 (6.43)	0.057	20.17 (5.83)	20.16 (5.90)	0.990
TLM (kg)	45.54 (6.94)	41.74 (5.39)	<0.001	34.33 (5.03)	32.42 (3.90)	0.014
ASM (kg)	19.79 (4.07)	18.14 (2.90)	0.003	14.23 (2.69)	13.64 (2.08)	0.148
hSMI (kg/m^2^)	7.25 (1.29)	6.93 (0.97)	0.079	6.13 (0.99)	5.92 (0.79)	0.100
wSMI (%)	29.13 (3.22)	29.25 (2.95)	0.812	25.53 (3.30)	25.06 (2.25)	0.343
HS (kg)	25.10 (11.40)	24.22 (7.89)	0.617	14.63 (7.50)	17.87 (4.90)	0.005

Except for *n*, values are given as mean (standard deviation). *n*: sample size; TBM: total bone mass; TFM: total fat mass; TLM: total lean mass; ASM: appendicular skeletal muscle (sum of the lean masses of the four extremities); hSMI: height-adjusted skeletal muscle index; wSMI: weight-adjusted skeletal muscle index; HS: handgrip strength.

## Supplementary Materials

The following are available online at https://www.mdpi.com/2077-0383/9/8/2333/s1, Figure S1: Age-based differences in two adjustment indices in a healthy control population. hSMI, height-adjusted skeletal muscle index; wSMI, weight-adjusted skeletal muscle index, Figure S2: Prevalence of low skeletal mass (Class I and Class II) using two adjustment indices in urban and community-based populations. hSMI, height-adjusted skeletal muscle index; wSMI, weight-adjusted skeletal muscle index, Table S1: Age-based differences in body composition, Table S2: Prevalence of low skeletal mass (Class I and Class II) in a healthy population using two adjustment indices.
