# Supplementary material for: Selecting Appropriate Sarcopenia Screening Methods for Asian Populations
Source: J Clin Med. 2020 Jul 22;9(8):2333. doi: 10.3390/jcm9082333 (PMC7464734; doi:10.3390/jcm9082333)
Supplement: Supplementary file 1 [file jcm-09-02333-s001.pdf]

Supplementary Table S1. Age-based differences in body composition

Age group (yr)	*n*	Weight (kg)	TBM (kg)	TFM (kg)	TLM (kg)	ASM (kg)	hSMI (kg/m^2)
Men	3028						
20-29	77	73.08 (11.20)	3.07 (0.43)	17.89 (7.74)	52.17 (5.03)	24.32 (2.68)	8.04 (0.82)
30-39	492	75.16 (12.78)	2.95 (0.43)	20.61 (7.71)	51.59 (6.32)	23.67 (3.11)	7.94 (0.86)
40-49	840	73.70 (10.49)	2.88 (0.39)	19.41 (6.58)	51.44 (5.37)	23.36 (2.90)	7.95 (0.83)
50-59	1014	71.73 (9.71)	2.81 (0.41)	18.69 (5.79)	50.26 (5.29)	22.33 (2.76)	7.79 (0.79)
60-69	481	70.01 (10.40)	2.77 (0.41)	18.32 (6.14)	48.86 (5.59)	21.47 (2.85)	7.62 (0.81)
70+	124	66.32 (8.74)	2.61 (0.43)	18.11 (5.53)	45.42 (4.77)	19.40 (2.52)	7.09 (0.72)
Women	2853						
20-29	134	55.70 (10.78)	2.30 (0.29)	18.24 (7.20)	35.02 (4.58)	15.28 (2.33)	5.92 (0.80)
30-39	556	55.47 (8.89)	2.30 (0.32)	18.38 (6.30)	34.64 (3.79)	14.82 (2.09)	5.73 (0.68)
40-49	822	57.15 (8.60)	2.29 (0.31)	19.65 (5.86)	34.91 (3.78)	14.78 (1.87)	5.83 (0.65)
50-59	889	57.86 (9.10)	2.14 (0.32)	20.84 (6.31)	34.53 (3.93)	14.37 (1.92)	5.83 (0.69)
60-69	347	57.61 (9.12)	1.97 (0.32)	21.08 (6.22)	34.11 (4.06)	14.10 (2.00)	5.89 (0.70)
70+	105	58.01 (8.81)	1.90 (0.34)	21.69 (6.19)	33.95 (3.80)	13.68 (1.80)	5.84 (0.65)

continued

wSMI (%)
33.65 (3.57)
31.82 (3.31)
31.93 (3.15)
31.30 (2.88)
30.86 (2.82)
29.37 (2.72)
27.73 (2.92)
26.93 (2.87)
26.08 (2.65)
25.07 (2.86)
24.69 (2.78)
23.81 (2.70)

Except for *n*, values are given as mean (standard deviation). *n*, number; TBM, total bone mass; TFM, total fat mass; TLM, total lean mass; ASM, appendicular skeletal muscle was calculated by summing the lean mass amounts of the four extremities; hSMI, height-adjusted skeletal muscle index; wSMI, weight-adjusted skeletal muscle index.

Supplementary Table S2 Prevalence of low skeletal mass (Class I and Class II) in a healthy population using two adjustment indices

	hSMI			wSMI		
	Normal	Class I	Class II	Normal	Class I	Class II
Men						
20-29 yr	87.0%	11.7%	1.3%	84.4%	11.7%	3.9%
30-39 yr	81.3%	15.7%	3.0%	73.0%	21.5%	5.5%
40-49 yr	83.1%	14.6%	2.3%	74.2%	22.3%	3.6%
50-59 yr	76.7%	19.7%	3.6%	67.4%	28.2%	4.4%
60-69 yr	68.6%	26.0%	5.4%	61.3%	32.4%	6.2%
70+ yr	45.2%	36.3%	18.5%	36.3%	51.6%	12.1%
Women						
20-29 yr	88.1%	11.9%	0.0%	85.1%	11.9%	3.0%
30-39 yr	83.8%	15.5%	0.7%	77.0%	19.2%	3.8%
40-49 yr	87.8%	11.9%	0.2%	69.3%	26.2%	4.5%
50-59 yr	87.2%	11.8%	1.0%	53.5%	36.2%	10.2%
60-69 yr	85.9%	13.5%	0.6%	49.0%	36.6%	14.4%
70+ yr	91.4%	8.6%	0.0%	32.4%	41.9%	25.7%

hSMI, height-adjusted skeletal muscle index; wSMI, weight-adjusted skeletal muscle index.

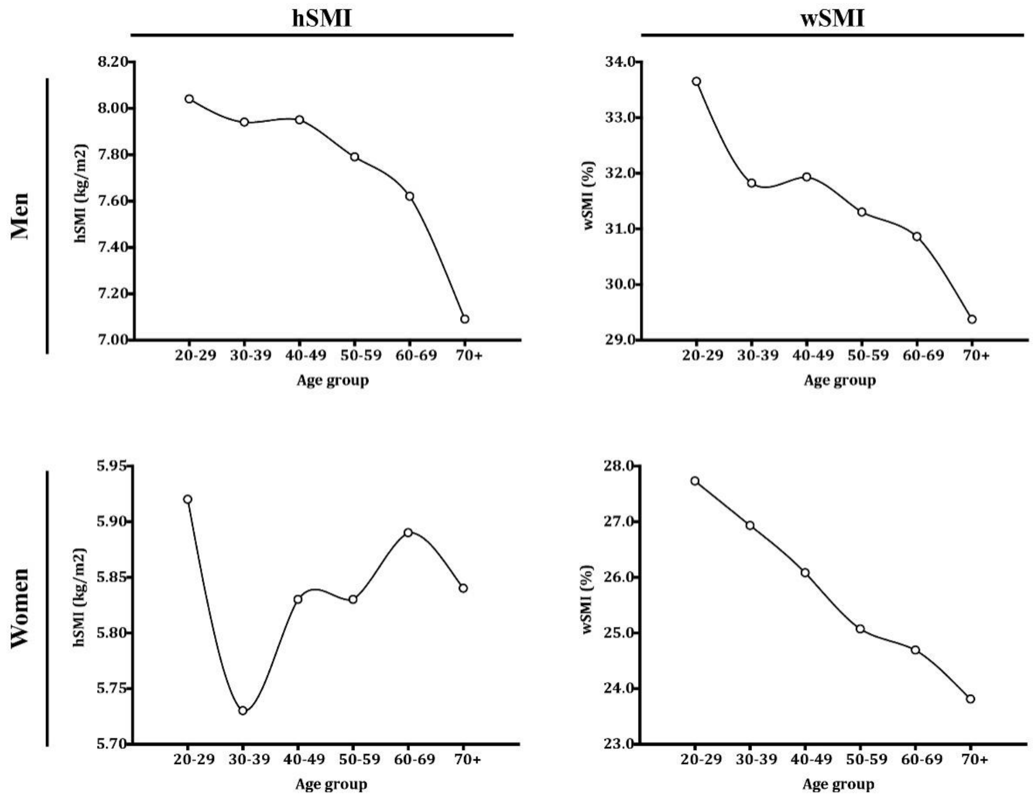

Supplementary Figure S1. Age-based differences in two adjustment indices in a healthy control population. hSMI, height-adjusted skeletal muscle index; wSMI, weight-adjusted skeletal muscle index

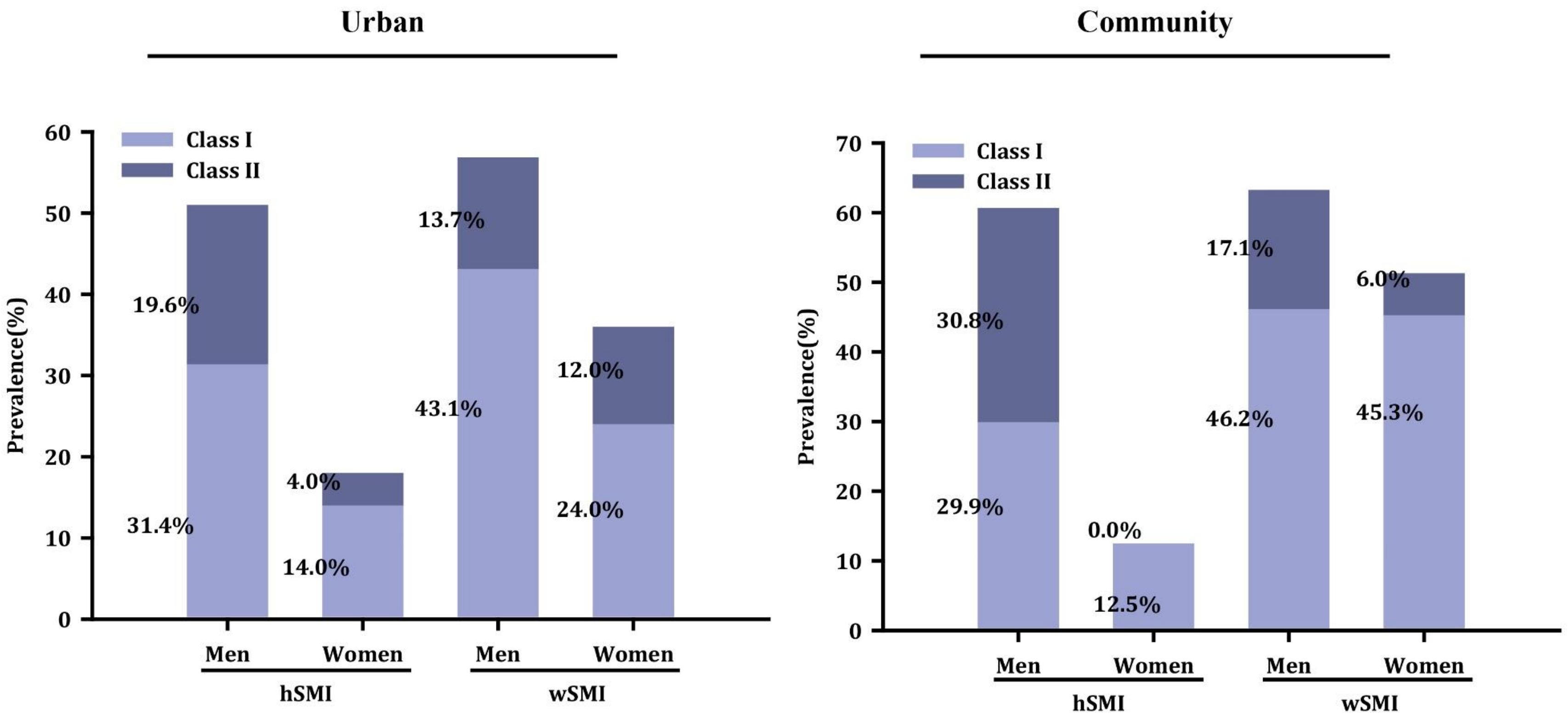

Supplementary Figure S2. Prevalence of low skeletal mass (Class I and Class II) using two adjustment indices in urban and community-based populations**.** hSMI, height-adjusted skeletal muscle index; wSMI, weight-adjusted skeletal muscle index
